# Inflammatory Myofibroblastic Tumors in Children: A Clinical Retrospective Study on 19 Cases

**DOI:** 10.3389/fped.2021.543078

**Published:** 2021-07-08

**Authors:** Min Da, Bo Qian, Xuming Mo, Cheng Xu, Haiyan Wu, Bin Jiang, Wei Peng, Jirong Qi, Jian Sun, Kaihong Wu

**Affiliations:** ^1^Department of Cardiothoracic Surgery, Children's Hospital of Nanjing Medical University, Nanjing, China; ^2^Department of Pathology, Children's Hospital of Nanjing Medical University, Nanjing, China; ^3^Department of General Surgery, Children's Hospital of Nanjing Medical University, Nanjing, China

**Keywords:** inflammatory myofibroblastic tumor, children, immunohistochemistry, recurrence, surgery

## Abstract

**Background:** Inflammatory myofibroblastic tumor (IMFT) is a rare neoplasm mainly affecting children and young adults. We conducted a retrospective study to evaluate the clinical features and treatment alternatives of childhood inflammatory myofibroblastic tumors.

**Methods:** A total of 19 patients who were pathologically diagnosed with IMT between December 2008 and October 2018 were included. Collected data were demographic information, main complaints, tumor characteristics, treatment, pathological results, immunohistochemical analysis, and prognosis.

**Results:** The male/female ratio was 13:6. The mean age at disease onset was 44.9 ± 33.9 months (range 4 to 111 months). The mean tumor size was 6.5 ± 4.0 cm (range 1.2 to 17.0 cm). The most common site was the abdomen (13/19). The most commonly used detection tool was CT. Eleven patients (57.9%) had aggressive tumor growth, including eight receiving extensive resection and three receiving palliative resection due to high local invasiveness and postoperative chemotherapy. Eight cases whose tumors were completely enveloped received complete resection. Immunohistochemistry was performed for 17 patients and ALK positivity was found in 11 patients. Despite three children lost to follow-up, sixteen patients were followed up for 6 to 132 months (average 63.9 months, median 66 months). Of which, twelve children survived with no evidence of IMT, and four cases (21%) showed local recurrences (two of them died). No distant metastasis was detected.

**Conclusions:** IMT is rare in children with various locations, mostly appearing in the abdomen. Whether the tumor could be completely removed, the location and the invasiveness of surrounding tissues might be highly prognosis-related.

## Introduction

Inflammatory myofibroblastic tumor (IMT) is a kind of mesenchymal tumor characterized with proliferation of myofibroblast spindle cells and prominent infiltration of plasmocytes and/or lymphocytes. IMT is rare and its true incidence and prevalence remains unclear (1). Commonly detected in the lung, retroperitoneal space, and pelvic and abdominal soft tissue, IMT usually attacks children and young adults. Pathologically, IMT tends to be locally invasive or recurrent, and rarely metastasizes (2, 3). Herein, we analyzed the clinical data of 19 children with IMT diagnosed at Children's Hospital of Nanjing Medical University. Literature was also reviewed to reveal its clinical features, imaging manifestations, diagnosis, treatment, and prognosis.

## Materials and Methods

The study analyzed the records of 19 patients confirmed as IMT at Children's Hospital of Nanjing Medical University between December 2008 and October 2018 were obtained. Collected data were demographic information, main complaints, tumor characteristics, treatment, pathological results, immunohistochemical analysis, and prognosis. This retrospective study was approved by the Medical Ethics Committee of Children's Hospital of Nanjing Medical University. Written informed consent was obtained from the [individual(s) AND/OR minor(s)' legal guardian/next of kin] for the publication of any potentially identifiable data included in this article.

Chi-square test (for categorical variables) was performed on SPSS 18.0. *P* < 0.05 was considered statistically significant.

## Results

The 19 children pathologically diagnosed with IMT, included 13 males and 6 females (average age 44.9 ± 33.9 months, range 4 to 111 months). Tumor locations varied largely, mostly in abdominal organs. Twelve cases showed abdominal distension, abdominal pain, vomiting, bloody stool, etc. Two cases with chest lesions and two cases with lung IMTs symptomized by cough, dyspnea and chest pain. One patient with tumor in chest wall presented local swelling and mass. One case with rectum polyp showed hematochezia. And one case of glottic IMT complained of hoarseness and difficult breathing at night. Despite three children who were lost to follow-up, sixteen patients were followed up for 6 to 132 months (average 63.9 months, median 66 months). These data were listed in Table 1.

**Table 1:** Demographic data and clinic characteristics.

**Case**	**Gender**	**Age (month)**	**Presentation**	**Location**	**Treatment**	**Recurrence**	**Follow-up**	**Status**
1	Female	35	Abdominal pain	Colon	Complete excision	None	131	Alive
2	Male	59	Abdominal mass	Abdomen	Palliative resection, chemotherapy	20 month after surgery	132	Alive with residual tumor
3	Male	110	Hoarseness, nocturnal, dyspnea	Glottis	Extensive excision	4 months after surgery	102	Alive
4	Male	6	Hematochezia	Colon	Extensive excision	-	lost	-
5	Male	27	Pulmonary mass	Lung	Complete excision	-	lost	-
6	Female	31	Abdominal pain	Omentum majus	Complete excision	None	82	Alive
7	Male	31	Emesis, abdominal pain, fever	Ileum	Extensive excision	None	78	Alive
8	Male	36	Abdominal mass	Omentum majus	Complete excision	None	77	Alive
9	Female	20	Abdominal pain	Colon	Complete excision	None	69	Alive
10	Female	57	Abdominal pain	Abdomen	Extensive excision	None	67	Alive
11	Male	5	Mass of chest wall	Abdomen	Extensive excision	None	65	Alive
12	Male	4	Abdominal distension, emesis	Small intestine	Extensive excision	None	62	Alive
13	Female	38	Abdominal pain	Omentum majus	Complete excision	None	51	Alive
14	Male	111	Cough	Thorax	Palliative resection, chemotherapy	1 month after surgery	13	Dead
15	Male	99	Hematochezia	Rectum	Extensive excision	-	lost	-
16	Male	57	Cough, fever, dyspnea	Thorax	Palliative resection, chemotherapy	1 month after surgery	6	Dead
17	Male	57	Abdominal pain, hematochezia	Colon	Extensive excision	None	40	Alive
18	Female	4	Emesis	Omentum majus	Complete excision	None	32	Alive
19	Male	66	Cough	Lung	Complete excision	None	15	Alive

CT was the most frequently used detection tool. Ten cases were confirmed with two or more imaging methods. CT was performed in 16 cases, ultrasound in 9 cases, colonoscopy in 1 case and electronic laryngoscope in 1 case. CT manifestations were often atypical, with patchy high-density shadows seen in heterogeneous masses. All patients underwent WBC and CRP examinations. Leukocytes and/or CRP (leukocytes increased in 4 children (14.27–25.99/L) increased in 10 children, and CRP increased in 7 children (12–179mg/L)]. Tumor markers (including NSE, AFP, CEA, CA199) were examined in 11 cases; NSE increased in 4 children, and AFP increased in 1 patient.

Tumor sizes ranged from 1.2 to 17.0 cm (average 6.5 ± 4.0 cm). All cases received surgery. In our series, fifteen tumors (78.9%) were localized within a single organ. Four tumors extended beyond a single organ at the time of presentation. In eleven cases showing invasive tumor, eight cases underwent extensive resection (tumor in one case showed ruptured capsule and implantation into the abdominal cavity), and other three cases extended beyond a single organ received palliative resection and postoperative alternative AVP regimen (Epirubicin + vindesine + cis-platinum complexes) or IEV regimen (etoposide + ifosfamide + leurocristine). Eight cases with encapsulated tumors received complete resection.

Tumors were round, oval or irregularly lobed in appearance, and the cut surfaces were gray, red, or dark red. Electron microscopy showed proliferation of spindle cells, mostly distributed in a bundle, and infiltration of inflammatory cells. Immunohistochemistry was performed in 17 patients, with 11 cases (11/17) demonstrating ALK reactivity. The results of all sample that test for Vimentin were positive (16/16), and all samples but one (16/17) were positive to varying degrees for Ki-67. The results of other markers were highly variable (Table 2).

**Table 2:** Immunohistochemistry results.

**Case**	**ALK**	**Vimentin**	**Dsemin**	**SMA**	**S-100**	**LCA**	**CD34**	**Ki-67**	**CK**	**CD68**	**CD117**
1	/	/	/	/	/	/	/	/	/	/	/
2	–	/	–	/	–	/	–	–	/	/	/
3	+	+	–	/	/	/	–	<20% +	–	/	/
4	+	+	–	/	/	/	+	1% +	±	+	±
5	+	+	±	/	/	/	–	10% +	–	–	/
6	/	/	/	/	/	/	/	/	/	/	/
7	+	+	±	/	/	/	-	5% +	+	+	–
8	+	+	±	/	/	±	–	15% +	–	+	–
9	+	+	±	/	–	–	–	10% +	±	±	–
10	+	+	–	/	–	–	–	15% +	±	+	–
11	–	+	±	/	–	/	–	10% +	–	+	–
12	+	+	+	/	–	/	–	25% +	±	+	–
13	–	+	+	/	-	/	-	±	/	/	/
14	+	+	–	+	–	/	–	20–70% +	–	+	/
15	+	+	±	+	–	+	–	30% +	±	+	/
16	–	+	±	+	–	–	–	75% +	/	–	/
17	–	+	–	/	–	–	–	±	/	–	–
18	+	+	+	+	–	–	–	10% +	+	–	–
19	–	+	+	–	±	–	–	10% +	+	/	/

Except for patients lost to follow-up, all four recurrences were observed in children (4/9) with aggressive tumors, and none in children (0/7) with intactly encapsulated tumors. Besides, recurrence rate was much lower in IMTs confined inside a single organ (1/12) than in those extending beyond a single organ (3/4) –*p* = 0.027). Glottic IMT recurring within 4 months after surgery was treated with reoperation and has been tumor free for 98 months with close follow-up. One case with lesions beyond the omentum to the intestine, bladder or colon at the time of presentation underwent incomplete resection and received postoperative chemotherapy, who was treated with several additional surgeries due to recurrences and has been close follow-up with residual tumor for 132 months. Incomplete resection was performed in two children suffering from thoracic masses which behaved aggressively and were closely adjacent to lung, pericardium, aorta, vena cava and other mediastinal structures. Both of them had regular postoperative chemotherapy, but both died of relapse at 6 and 13 months after surgery, respectively.

## Discussion

IMT is a unique tumor that can exist in any anatomical site and has affinity for children's lungs, soft tissues and abdominal organs. IMT may arise from infectious diseases, abnormal repair, EB virus or special bacterial infections, excessive inflammatory reactions after surgery, other malignancies or autoimmune diseases (4). However, little research is available to support or refute any of the above etiologies. The mechanism and pathogenesis of IMT remains elusive.

The gender ratio varies in different cohorts in previous reports and male predominance was found in our cohort (male: female, 13: 6) (5–7). Kovach (8) and Tutku Soyer (7) had reported that lung is the most common site of IMT. Bute in our cohort, IMT was mostly found in the abdominal parts (13/19), including intestine (7 cases), omentum (4 cases), and left upper abdomen (1 case). The other case showed metastasis from the omentum to the intestine, bladder or colon at the time of presentation. This result is consistent with that reported by Dalton et al. (9).

The clinical symptoms of IMT are usually organ-specific. CT examination is the most reliable diagnostic method. CT examination was performed before operation in 16 children, which can guide the formulation of surgical plan. The results of laboratory tests vary widely. Ten children showed increased leukocytes and/or CRP, which returned to normal after surgical resection, except in two of them undergoing palliative surgery. Therefore, these lab tests are not reliable for preoperative diagnosis of IMT, but perhaps for predicting its recurrence. Tumor markers we tested are also not specific to IMTS.

The final diagnosis still depends on pathological and immunohistochemical results. Approximately 50% of IMTs carry ALK gene rearrangement and overexpressed ALK. In our patients, 11 cases were immunohistochemically ALK-positive 57.9% (11/19), and this proportion is similar to that mentioned in the primary reports (5). Antonescu et al. (10) found that more than 90% of children with IMT demonstrated gene rearrangement (ALK gene, ROS1 gene, RET), which provides a possibility of developing targeted treatment of IMT. In recent years, ALK inhibitors such as crizotinib have achieved promising results in the treatment of IMT (11–13). But so far, complete surgical resection remains the first choice (14, 15). Craig et al. (16) recently reported a group of cases with ALK-positive IMTs that received ALK inhibitor treatment before or after surgery and obtained a curative response. All of our patients underwent surgical treatment. Sixteen children received completely resection, while three cases received palliative resection due to local invasion to adjacent tissues and organs and subsequent postoperative chemotherapy.

Characterized by its rare local recurrence and metastasis, IMT is considered moderately malignant and has favorable prognosis (17–19). In the initial surgery, complete excision of tumors with clear surgical margins can prevent recurrence (9). The recurrence rate varies with lesion site, from <2% in tumors confined to the lung to 25% in extrapulmonary lesions, such as multinodular intra-abdominal tumors in delicate anatomical organs (e.g., larynx or trachea) and distant metastasis is rare (2, 20). Excluding three cases lost to follow-up, long-term survival of twelve patients was achieved in our cohort and none of the cases receiving complete resection experienced recurrence. One of the patients received extensive excision relapsed. The recurrence might due to residual tumor tissue at surgical margin during primary surgery since the procedure was difficult to perform at larynx. Secondary extended resection under electronic laryngoscope was taken immediately since revealed, and no evidence of further recurrence was found for 98 months. Other three recurrences were found in patients receiving palliative resection. The patient displayed massive invasion of recurrent IMT in the abdominal cavity, and intestinal obstruction during regular chemotherapy at 20 months after primary surgery; palliative surgery was performed to release the symptom. The patient received common bile duct interventional dredging due to the invasion into the common bile duct at 2 years and 3 years, respectively, after the second operation and common bile duct drainage at 6 years after the second operation. In addition, this child was closely followed up for 132 months despite with poor quality of life.

The other two patients diagnosed with thoracic IMTs exhibited serious local invasiveness to lung, pericardium, aorta, vena cava and other mediastinal structures, and received common chemotherapy after incomplete surgery. Recurrence was both observed at the one-month follow-up after surgery. One of them refused further treatment, because the family could not afford the expenses, and died of secondary airway obstruction at postoperative Month 6. The other refused reoperation, kept on chemotherapy and other adjuvant therapy and died of heart failure secondary to tumor compression at postoperative Month 13. Of all recurrences, only one was confined to a single organ and three extended beyond a single organ. Our results suggest that whether the tumor could be completely removed, the location of the tumor, and the extent of tumor invasion of surrounding tissues might be related to recurrence and death. Chemotherapy seems to achieve little after palliative surgery in patients with serious local invasiveness. No distant metastasis was detected in all cases.

In summary, we reported 19 children with IMT in this study. The tumor locations vary, and the most common site of tumors in our series was the abdomen. Pathological and immunohistochemical tests are the gold standards for IMT diagnosis. CT can help define tumor anatomy and guide surgery. Complete surgical resection is preferred and can limit the recurrence. But whether the tumor is completely removed or not, tumor location and invaded area might be related to recurrence and death. Chemotherapy seems to achieve little after palliative surgery in patients with serious local invasiveness and further studies are needed to detect more promising treatment options.

## Data Availability Statement

All datasets generated for this study are included in the article/supplementary material.

## Ethics Statement

This retrospective study was approved by the Medical Ethics Committee of Children's Hospital of Nanjing Medical University.

## Author Contributions

XM designed the study and helped write the manuscript. MD performed the experiments, interpreted data, and wrote the manuscript. BQ collected and analyzed data. CX analyzed data. HW, BJ, WP, JQ, JS, and KW interpreted and analyzed data.

## Conflict of Interest

The authors declare that the research was conducted in the absence of any commercial or financial relationships that could be construed as a potential conflict of interest.
